# Care continuity, team autonomy and resource planning systems in relation to perceived time pressure among Finnish home care nurses: a cross-sectional multi-source study

**DOI:** 10.1186/s12912-025-04028-2

**Published:** 2025-10-30

**Authors:** Salla Ruotsalainen, Visa Väisänen, Laura Corneliusson, Tiina Pesonen, Juhani Sulander, Timo Sinervo

**Affiliations:** 1 Finnish Institute for Health and Welfare, Welfare State Research, Processes and Policies Team, Mannerheimintie 166, Helsinki, 00330 Finland; 2 Faculty of Social Sciences, Health Sciences, Tampere University, Tampere, Finland; 3 Department of Health and Social Management, Faculty of Social Sciences and Business Studies, University of Eastern Finland, Kuopio, Finland; 4 Department of Nursing, Umeå University, Umeå, 90187 Sweden

**Keywords:** Home care, Time pressure, Team autonomy, Care continuity, Practical nurses

## Abstract

**Background:**

Due to a growing number of older people, nurses working in Finnish home care have been subject to increasing efficiency requirements leading to e.g., growing job demands, and turnover. Previous research has indicated that factors such as care continuity and self-organizing teams are beneficial to staff wellbeing and care quality, however, the research incorporating workday characteristics remains scarce. The aims of this study were to examine (1) how individual level work characteristics and job demands are associated with time pressure among home care nurses, and (2) how time pressure varies between work organization factors at the organizational level.

**Methods:**

A cross-sectional wellbeing survey for home care nurses in 16 teams, across Finland (*n* = 416). Further, items from a managers’ survey and RAI data sources were merged with the survey data. Linear regression analysis was used to analyze time pressure and individual level variables. T-tests and ANOVA were used to analyze the differences in time pressure among organizational level variables.

**Results:**

Lower proportion of breaktime, lower care continuity, working alone, and if something disrupted the course of a workday were associated with higher perceived time pressure. Of the organizational level factors, teams with higher autonomy and an Enterprise Resource Planning (ERP) system that took teams into account when planning client visits perceived lower time pressure.

**Conclusions:**

Flexibility is needed in home care nurses’ workdays. If an ERP system is used, it should ensure care continuity and allow for changes if disruptions during the workday occur. Due to the independent nature of home care work, collegial support is needed to cope with difficult situations. Furthermore, home care teams should have more autonomy over their work, which might lead to higher job satisfaction and retention among staff.

## Introduction

The lack of care workforce is a global phenomenon. Especially in Finland, where the population is very aged and still aging rapidly [1], ensuring the availability of care staff will be even more challenging in the future. In Finland, institutionalized care as a form of service for older people has been phased out and transformed into assisted living facilities with 24-hour assistance, with home care acting as the current priority care option [2]. Home care is formed of home help services and home nursing, including care and caring services that support function and nursing related issues [3]. However, the current Finnish home care is more focused on the basic care of the client. Housekeeping tasks, for instance, are not included in the work tasks of the nurses working in home care, and consequently, if a client needs help with cleaning, they may obtain the services via home care provider, with possible out-of-pocket costs. On the contrary, residents in assisted living facilities live in their own apartments with care staff present either round the clock or during the day. All care and other services, excluding specialized care, are received from the facility. While approximately half of the assisted living facilities are privately owned, home care is mainly provided by the public sector. The majority of home care staff are licensed practical nurses with three years of vocational training. While the amount of older people receiving home care has grown, the number of care personnel has not increased accordingly, creating problems among current home care employees, such as stress and turnover intentions [4]. When comparing Finland to other Nordic countries, home care is perceived as a highly stressful work environment, exemplified by sleep problems caused by work being more common in Finland than in other Nordic countries [5].

The current care policy that prioritizes home care includes an aim of having the amount of direct care time (the time spent directly in contact with a client) to be approximately 60% of the total workday among practical nurses [6]. In the Finnish home care services, client visits are often scheduled using Enterprise Resource Planning (ERP) systems, in order to optimize the routes and tasks aimed to be conducted with the client. They are often further used for managerial purposes, for instance tracking the workforce situation and presenting client-related information. Criticism towards ERP systems has also emerged: A previous study found that due to the use of ERP systems, the clients were rarely seen by a same nurse, indicating low care continuity, with additional problems manifesting since the work planning was centralized with no direct contact to the home care teams [4].

Time pressure is shown to be prevalent especially among nurses working in older people care [7, 8]. If nurses must constantly work with a feeling of not having enough time for clients or for completing their tasks properly, severe implications for the quality of care are likely [9, 10]. In addition, time pressure, among other job demands like moral distress, have been associated with lower general psychological wellbeing and job satisfaction [11] as well as higher turnover intentions [12]. Moreover, high job demands have been further linked with higher incidence of sickness absence among care workers [13]. Previous research has further shown that already busy schedules of home care nurses are worsened by sudden disruptions, potentially leading to more mental and physical health problems [14]. These factors stress the importance of further examining the characteristics associated with time pressure, to both identify the aspects that may assist in better planning and work distribution, and to investigate how to best increase job satisfaction and workforce retention among nurses.

An important organizational factor identified as especially meaningful for care work is care continuity. Care continuity can have multiple meanings depending on the context, for example informational continuity or management continuity [15]. In this study, we examine care continuity from the perspective of consistency in the relationship between the care worker and the client [16], referred to as relational continuity [15]. We assume that a smaller number of different clients indicates higher care continuity from the care worker’s perspective, with the number of total visits remaining constant. In many cases, large teams (e.g., more than 12 nurses), high staff turnover, or work planning not considering the team structures, (instead purely allocating staff based on path efficiency or resource optimization), can negatively affect the continuity of care. In these situations, a high number of different nurses may visit a single client, leading to low care continuity, especially among those with a need for high frequency care visits [17, 18]. This might further result in fragmented and impersonal care [19], potentially compromising the quality of care as well as increasing the time pressure and stress, and decreasing the job satisfaction of nurses. Russel and colleagues [20] demonstrated in their study that clients were less likely to be hospitalized and to use emergency care services when consistency in nursing personnel (i.e., care continuity) was higher. In home care, clients with higher personnel continuity have had fewer depressive symptoms and falls [21]. In addition, higher care continuity has led to home care nurses being more likely to remain employed [22], in addition to experiencing higher job satisfaction [7]. Notably, even if the nurses might have a high number of clients during the workday, the number of *different* clients can be an important indicator of care continuity in home care, and higher frequencies of different clients may result in increased time pressure due to the nurses needing to familiarize themselves with the clients documentation and preferences to ensure high quality care. The visits of clients requiring multiple visits per day could be done by the same nurse in one shift, which would be beneficial from the perspective of care continuity and consequently indicate better a nurse-client relationship.

Self-organizing teams have been shown to have several benefits for care work in older people services [23, 7]. Teams that are able to, for instance, distribute their client visits autonomously can better ensure the continuity of care, especially if the teams are small enough. Smaller teams can ensure that nurses are able to work with familiar clients, simultaneously matching the length of visits in accordance with the care needs of the client [24]. Furthermore, recent research has shown that working in self-organized teams benefits employee satisfaction and wellbeing. Nurses in self-organizing teams have more autonomy over the care of their clients, which has led to higher job satisfaction [25], and they have been able to work with a familiar set of clients, ensuring higher care continuity [26]. An earlier study [23] showed that strain (the situation when job demands, e.g. time pressure, is high and autonomy low) mediated the effect of team autonomy on job satisfaction and turnover intentions. We hypothesize that if the nurses have the possibility to plan their work and have enough flexibility when needed, perceived time pressure could be lower as well.

However, the conditions in which self-organized teamwork works and how other organizational factors affect self-organized teamwork are unknown. The implementation of self-organized teamwork might be challenging, as changing the hierarchy and management might be difficult, in addition to ensuring the sufficiency of employees’ teamworking skills [27]. In addition, there might be planning systems which contradict autonomous work planning in teams. As, at least in Finnish home care, the use of ERP (enterprise resource planning) systems is highly prevalent and recommended from the ministerial level [2], it is important to ensure that ERP systems and self-organized teams can work together. Yet, this may not be realized, especially if the ERP systems do not account for the team structures when planning the client visits. Often ERP systems prioritize efficiency by selecting the shortest distances to destinations, rather than considering the existing team structures and linking nurses to their own clients (or prioritizing care continuity). Frequently, employees criticize the ERP systems as non-responsive, as it may difficult to make changes to the work list if something unexpected happens or if the team would prefer to organize their visits differently [28]. ERP systems may also allocate an insufficient amount of time for care tasks, as well as not take into account unexpected events or team structures, potentially leading to increased perceived time pressure.

Retention of nursing workforce is a key issue for both policy and practice in the upcoming years, as the numbers of older people is expected to increase [29]. Since home care is usually characterized as being highly independent work, having to work without adequate support or collegial help might be straining for the employees [13]. A Norwegian study among home care workers demonstrated that even if the nurses worked independently, they often received support from their colleagues and were able to call them if needed. This support was one important factor which increased their intentions to stay in home care [22]. 

In home care, factors relating to the ways how work is organized, such as time management and task distribution, have been associated with work ability and occupational wellbeing [30]. Similarly in Finnish assisted living services, workday characteristics, namely the higher number of care events and the care needs of clients, have been associated with lower care worker wellbeing [31]. Time pressure and other work organization related factors, such as unplanned additional tasks and delays, were identified as being especially burdening for home care staff in a study by Gebhard & Wimmer [32], whereas increased flexibility of work schedule has been linked with lower intentions to leave among nurses in the hospital sector [33] and in home care [22]. In regard to more objective job demands, or how workdays are comprised, a review by Wendsche and colleagues [34] demonstrated that in the presence of high workload and job demands, breaks were more often missed or skipped. While the same review further indicated that more breaks were associated with higher wellbeing and lower turnover intentions, some studies in the review did not find any associations between improved wellbeing or retention and breaks [34].

Previous studies have mainly investigated time pressure as a stressor, rather than as an outcome. Earlier studies have attempted to determine the association between job demands or time pressure, and negative outcomes, for example poor job satisfaction or turnover, but they have mainly relied on single surveys, instead of combining several sources of information. Factors related to how workdays are comprised, or indirectly measured job demands, have been scarcely studied in relation to time pressure. Moreover, not much is known about the relationship between care continuity and time pressure. Further evidence on these factors can be utilized to both improve job satisfaction and wellbeing of nurses, the quality of care, and consequently support the recruitment and retention of care workforce. The aim of this study was first, to examine on the individual level which work characteristics and job demands are associated with time pressure among home care nurses, and second, to examine how time pressure varies between different work organization factors at the organizational level. Based on the literature, we hypothesize that both individual and organizational level factors, especially reduced job demands and more flexible work organization (team autonomy, care continuity), positively affect the perceived time pressure among nurses working in home care.

## Methods

This study is a part of a larger Time Measurement project that was conducted in work units providing care for older people in several locations in Finland in 2021. The project aimed to obtain information related to the amount of care time the clients in different care facilities receive as well as information regarding work tasks performed with clients, simultaneously considering the clients’ care needs. The study was cross-sectional in nature with 17 participating home care units.

### Participants and data collection

In our sample, we included licensed practical nurses and registered nurses who worked in the units that participated in the study. To be included in our final sample, the minimum number of client visits during the work shift required was three. This was done to limit the participants to those working in client care and not for example in more administrative duties.

A brief wellbeing survey was sent by mail together with the time measurement form to all the employees in the home care units that participated in the study. Units which were part of the Finnish Resident Assessment Instrument (RAI) network were invited to the study. The invitation was sent by e-mail via managers of older people care in the wellbeing services counties. The units that participated in this study (*n* = 17) were located in four different wellbeing services counties (out of 22) in Southern and Central Finland. The number of surveys sent was based on the managers’ estimation of the number of employees working daily in the units (*n* = 416). The number of employees was an estimate as the workforce situation in home care units can fluctuate due to sickness absences, vacations, and poor temporary workforce availability. The inclusion criterion was employment (permanent or temporary) in the participating units. In total, 384 nurses returned the surveys. The data collection took place in October 2021.

The survey, previously used in studies by Kaihlanen, et al. (2023) and Väisänen, et al. (2024), included two parts: The first part was the time measurement form, where workers filled in their work tasks done during the day (one task per line). Start and end times, task category (e.g., helping with hygiene or medication) and the name of the client (if applicable) were documented. The forms were filled for one work week, which was a part of their work tasks during the measurement week. The second part of the survey was a short wellbeing questionnaire. This was completed once, after the first workday of the time measurement period, and responding to this was voluntary. In addition, we used data from an online survey that was sent to managers of the participating organizations. The survey included information about organizational characteristics, such as the number of clients and functioning of teams. This organization-level data was supplemented by the individual-level survey data (aggregated). Furthermore, to obtain information about clients’ need for care, data from the Resident Assessment Instrument (RAI) was merged with the time measurement and wellbeing surveys by client names. The Resident Assessment Instrument (RAI) includes data based on validated instruments, describing for example the client’s health status.

### Measures

The dependent variable of Time Pressure was formed of two questions where a person was asked to assess if the following had disturbed, worried, or strained them today: “*I have too little time for my patients/clients*”, and “*I do not have time to perform my work properly*”. The questions were adapted from the Nurse Stress Index by Harris [35]. The answers were rated on a five-point Likert Scale (1 = ‘Not at all’ to 5 = ‘Very much’). Spearman-Brown coefficient for the two items was 0.84.

The indirectly measured job demands included the number of different clients per workweek, which to account for varying number of workdays per week was standardized to five workdays per week. The variable was used as an indicator of care continuity, where a smaller number of different clients indicated better care continuity, referring to a continuity or consistency in a relationship between the care worker and the client [16]. The number of different clients has been used as an indicator for care continuity in previous studies, in similar settings [7, 36]. Compared to the total number of clients per day, the number of different clients better represents care continuity, as the total number can include several visits for same client, and therefore does not provide adequate information on care continuity or consistency related to clients. The proportion of care time consisted of both direct and indirect care time. This included care work that was related to clients and was done either in direct (e.g., daily hygiene or aiding with eating) or indirect contact (e.g., care planning, contacts with client’s next of kins) [37]. The proportion of care time and the proportion of breaktime were measured as a percentage of the total workday. Last, the Case Mix Index (CMI) was used to determine the clients’ need for care. The index is based on the Finnish RUG-III/18- classification, obtained from RAI assessments [18]. It indicates how much resources each client requires compared to an average client (value of 1.0). For example, a client with a CMI value of 1.2 needs on average 20% more care resources, and a client with a CMI value of 0.8 needs on average 20% fewer resources. The mean CMI-value was calculated from the nurse’s workday’s clients.

Further two individual level variables related to work characteristics from the wellbeing survey were ‘distress related to lack of help and consultation possibilities’ (from now on referred to as ‘working alone’) [4, 38] and an assessment of whether something disrupted the course of the workday. Working alone was rated on a five-point Likert Scale (1 = ‘Not at all’ to 5 = ‘Very much’). The item measuring disruptions during the workday was posed as “Assess today’s workday,” and the answer options were “went as planned” (= 0), “went nearly as planned” or “something disrupted the course of the workday”. The last two options were merged as ‘something disrupted the workday’ (= 1).

In addition to individual level variables, this study included organizational level independent variables that were related to the organization of work. First, team autonomy, as assessed by supervisors, was measured as a sum variable formed of seven items with a Cronbach’s Alpha of 0.77. It included statements about whether the teams were able to make decisions regarding work shifts, client visits, recruitment of new employees, use of substitute workers, working methods, care of clients, and participation in training. These questions were rated on a four-point Likert Scale from 1 = ‘Not at all’ to 4 = ‘Team is able to decide autonomously’. The units were grouped in three, based on the level of autonomy: low (≤ 2.5), medium (> 2.5 to < 3.0), and high (≥ 3.0).

Two questions related to ERP system were used: “*an Enterprise Resource Planning system (ERP) takes into account the care teams when planning the client lists*” (1 = ‘Yes’ and 0 = ‘To some extent) and “*Are teams able to alter the lists created by ERP system?*“ (1 = ‘Yes’ and 0 = ‘To some extent’). ERP systems taking care teams into account can mean that, for example when creating the list of client visits, the team structure is maintained and nurses are matched with their own clients, even if this disrupts the travel optimization. Last, an open-ended question asking to describe the current care workforce availability and the substitute situation was coded as “Challenging” or “Good” based on if the managers felt they had difficulties with obtaining staff. Two researchers from the research group performed the ranking and afterwards the interpretations were discussed and modified accordingly. Challenging workforce availability for example meant that there were empty vacancies and no (qualified) applicants for the job, while good workforce availability meant no issues filling empty vacancies and that units had mainly permanent staff working in teams.

Background variables included age, female sex (reference: male), and registered nurse (reference: practical nurse), which were used for adjusting in the analyses.

### Data analysis

Descriptive analyses were used for describing the data. Next, we analyzed the individual level data using univariate and multivariate linear regression, and last, comparisons of group means (T-Test and ANOVA) were used to analyze the association of organizational level variables related to work organization to time pressure. Since the individual level data was clustered at the home care unit level, the clustered standard errors were adjusted for in the regression models. This provides more accurate confidence intervals and p-values, in addition to correction of the assumption of independence of observations [39]. Furthermore, since variables related to ERP system and team autonomy only varied on a team level, we chose to use T-test to analyze the differences between time pressure and aforementioned variables. To examine the possibility that the differences occur due to differences in clients’ care needs, we further conducted T-tests and ANOVA for the organizational variables and the care needs of clients (mean CMI).

The linear regression analyses were built in three steps. First, univariate analyses were conducted with all the independent variables and the dependent variable (time pressure). Second, multivariate analyses were performed by including all the indirectly measured job demands (proportion of care time and breaks, number of different clients / care continuity and the mean CMI of the clients, i.e., care needs). Last, we included subjective job demands (strain related to working alone and if something had disrupted the course of the workday) into the model. The multivariate models were adjusted for age, sex, and occupational status (practical vs. registered nurse). Missingness was checked by examining whether the participants with missing and non-missing values differed from each other. Those with missing values in time pressure had on average more weekly different clients (24.0 and 32.6, p = < 0.001) and lower proportion of breaktime (6% and 3%, p = < 0.001) than those with non-missing values. Other independent variables did not statistically differ between the two groups. This may indicate that participants with missing values in time pressure had busier work schedules compared to those with valid values. In total, 67 participants (6%) were not included in the regression analyses due to missing values in the outcome variable. Missingness in other variables ranged between 0 and 71 (mean = 26.6), and missing values were handled using pairwise deletion. To determine the appropriate sample size for this study, a reference population of 20,100 individuals was considered, corresponding to the estimated number of personnel working daily in home care services across Finland [40]. Based on a statistical power of 95% and a significance level of 0.05, the minimum required sample size was calculated to be 377 participants.

The analyses were conducted using SPSS, version 29, and R Statistical Software version 4.2.2 (R Core Team, 2020). The alpha level was set to 0.05.

## Results

### Sample characteristics

The total number of participants was 384, with an approximated response rate of 92%. The majority of the respondents were licensed practical nurses (*n* = 340, 89%). The mean age of the participants was 43 years (SD 13.0). The mean value for time pressure was 2.3 (SD 1.1). The characteristics of the respondents are described in more detail in Table 1.

Of the organizational level variables, the mean for team autonomy was 2.75 (SD 0.45), indicating a medium level of autonomy. Care workforce availability was stated to be “good” in 44% of the units. In almost half of the units (46%) teams were able to alter the plans of an ERP system and in 38% the ERP systems took teams fully into account when planning the client visits.

**Table 1:** Sample characteristics by occupational status

Variable	Registered nurses *n* = 44		Practical nurses *n* = 340		All *n* = 384	
	Mean	SD	Mean	SD	Mean	SD
Age	44.0	10.7	43.3	13.3	43.4	13.0
Sex (female, %)	100%		91%		92%	
Time pressure (1–5)	2.28	1.06	2.29	1.11	2.29	1.10
Proportion of care time (%)	42%	15%	51%	11%	50%	12%
Proportion of breaktime (%)	5%	3%	5%	4%	5%	4%
Number of different clients during the week	15.3	9.0	19.1	8.2	18.6	8.3
Mean CMI^1^-value of clients	0.95	0.13	1.10	0.24	1.09	0.23
Distress due to working alone (1–5)	1.68	0.97	1.64	1.05	1.65	1.04
Something disrupted the course of the workday (yes, %)	84%		63%		65%	

^1^ Case Mix Index. Indicating how much resources each client requires compared to an average client (value of 1.0)

### Univariate associations

The results of the univariate analyses showed that higher proportions of care time, higher number of different clients (care continuity), lower proportions of breaks, higher distress related to working alone, and if something had disrupted the workday were associated with higher time pressure (Table 2). The care needs of clients (CMI) were not associated with time pressure.

**Table 2:** Linear regression analysis on the effects of individual level job demands and workday characteristics on time pressure (*n* = 384)

Adjusted for clustered standard errors (standardized betas)									
Variable	Univariate			Multivariate 1			Multivariate 2		
	β	95% CI	*p*	β	95% CI	*p*	β	95% CI	*p*
Age	**0.21**	**0.09–0.33**	**< 0.001**	**0.18**	**0.06–0.29**	**0.003**	0.11	-0.01–0.24	0.067
Female (ref: male)	0.18	-0.20–0.56	0.351	0.09	-0.22–0.41	0.551	0.08	-0.20–0.35	0.582
Registered nurse (ref: practical nurse)	-0.01	-0.34–0.32	0.954	0.06	-0.39–0.51	0.783	-0.13	-0.44–0.18	0.422
Proportion of care time	**0.14**	**0.05–0.24**	**0.004**	0.06	-0.05–0.18	0.281	0.03	-0.06–0.12	0.467
Proportion of breaktime	**-0.18**	**-0.29– -0.07**	**< 0.001**	**-0.13**	**-0.25–0.00**	**0.050**	**-0.13**	**-0.25– -0.00**	**0.042**
Number of different clients during the day (weekly average)	0.18	-0.02–0.38	0.083	**0.27**	**0.11–0.42**	**< 0.001**	**0.15**	**0.02–0.28**	**0.022**
Mean CMI^1^-value of clients	-0.07	-0.20–0.07	0.320	-0.08	-0.18–0.03	0.159	-0.02	-0.14–0.10	0.794
Working alone (1–5)	**0.34**	**0.26–0.42**	**< 0.001**				**0.26**	**0.18–0.34**	**< 0.001**
Something disrupted the course of the workday (yes)	**0.93**	**0.67–1.19**	**< 0.001**				**0.74**	**0.51–0.97**	**< 0.001**
				R2 = 0.096. DF = 278			R2 = 0.300. DF = 258		

^1^ Case Mix Index. Indicating how much resources each client requires compared to an average client (value of 1.0)

### Multivariate associations

In the multivariate models with individual level job demands, only having a higher number of different clients was associated with higher time pressure. Standardized beta coefficients, confidence intervals and p-values of the models are presented in Table 2. In the final model with job demands and work characteristics related variables, lower proportion of breaks and higher number of different clients were associated with higher time pressure. In addition, working alone and something disrupting the course of the workday further contributed to the model statistically significantly. In total, the final model seemed to explain perceived time pressure rather well (adjusted R-square 0.30).

### Work organization and time pressure

The results of the analyses related to work organization and time pressure on the organizational level are presented in Table 3. The results showed that those nurses who worked in teams where an ERP system takes teams into account when planning the client visits had lower time pressure. In addition, if an ERP system allowed teams make changes into the lists, the nurses had slightly lower time pressure. However, this difference was not statistically significant (*p* = 0.07). Furthermore, nurses working in more autonomous teams perceived on average lower time pressure. The differences in mean time pressure were statistically significantly different between the most autonomous and the two other groups (*p* = 0.011 for the difference between highest and medium and *p* = 0.010 for the difference between highest and lowest). We found no significant difference in time pressure between care teams with high workforce availability and low workforce availability.

The results of the analyses investigating differences in clients care needs and organizational level factors showed that those in teams that allowed for altering the plans of an ERP system had on average clients with slightly higher care needs (CMI value 1.13 vs. 1.07, *p* = 0.032). In addition, clients’ average care needs varied according to team autonomy level, but the difference was significant only between the lowest autonomy and medium autonomy groups (CMI value 1.15 vs. 1.03, *p* = 0.001). High autonomy group had a mean CMI value of 1.10. Lastly, nurses in units with high care work availability had on average clients with higher care needs (CMI value 1.16 vs. 1.03, *p* > 0.001). To summarize, the care needs of clients seemed not to explain the differences between work organization factors and perceived time pressure.

**Table 3:** Analysis of work organization related variables and time pressure on the organizational level (*n* = 17)

Variable	Time pressure	Tests for group differences	
	Mean (SD)	T-value / F-value	p
ERP^1^ system takes care teams into account: Yes	**1.99 (1.18)**	**3.37**	**< 0.001**
ERP system takes care teams into account: To some extent	**2.43 (0.96)**		
ERP system allows teams to make changes: Yes	2.14 (1.21)	-1.79	0.074
ERP system allows teams to make changes: To some extent	2.38 (1.00)		
Team autonomy: High	**2.05 (0.99)**	**6.38**	**0.002**
Team autonomy: Medium	**2.53 (1.17)**		
Team autonomy: Low	**2.46 (1.13)**		
Care workforce availability: Good	2.20 (1.18)	1.30	0.195
Care workforce availability: Challenging	2.37 (1.03)		

^1^ Enterprise Resource Planning system

## Discussion

The aim of this study was to examine which individual level job demands and work characteristics are associated with time pressure among home care nurses. In addition, we aimed to investigate the relationship between work organization factors and time pressure on the organizational level. The results of this study indicate that higher number of different clients, i.e., lower care continuity, lower proportion of breaks, distress due to working alone, and disruptions were associated with higher perceived time pressure among home care nurses. In addition, the analyses of work organization factors and time pressure indicated that time pressure was lower among nurses working in care teams with higher autonomy, as well as in care teams with an ERP system that considered the care teams.

In the univariate model, we found an association between higher amount of care time and higher time pressure. However, the association lost its significance in the multivariate model. Our findings further showed an association between lower care continuity (i.e., high number of different clients) and higher time pressure. This may indicate that a higher number of unfamiliar clients during the work week is of more importance to time pressure than just the amount of care time conducted itself. This could suggest that the workdays may be perceived as busier when an employee has a lot of different and unfamiliar clients, compared to having an equally busy workday but with more familiar with clients, potentially with daily repeat visits. With a higher number of different more unfamiliar clients, the nurses must orient themselves to a new client and their home, and potentially spend more time studying the nursing documentation. These activities might take less time or need not be done if the client is familiar to the nurse. Previous research has found positive outcomes of care continuity, for example care continuity has been associated with higher intent to remain employed [41] and better job satisfaction [7] among home care employees. Better care continuity has been further associated with reduced emergency visits and hospitalizations among clients [20], which could reflect familiar nurses being more able to detect shifts in a client’s condition. When considering the client’s perspective, benefits of care continuity, such as feeling more secure and provision of higher quality of care, have been identified [42]. For home care nurses, better continuity of care might alleviate the perceived time pressure. If a nurse can work with a smaller pool of clients, the work might become more seamless as interactions with clients and the environments are more known and familiar. A familiar environment might also save time in a nurse’s workday if a nurse does not have to familiarize themselves with every client and their documentation, even with a busy schedule. A nurse knows where things are situated in a familiar client’s home, therefore possibly leading to more efficient and faster work. These factors might therefore have a positive influence on time pressure. An important factor related to the quality of care and care continuity is that changes in a client’s conditions might be easier to detect when a client is familiar to a nurse, and in some cases a familiar nurse might notice conditions that require more urgent care [22]. Supporting care continuity may also enable staff to work in a person-centered manner, which has been associated with positive outcomes for both clients and care staff [43]. In addition, the clients do not have to explain their situation repeatedly to different nurses, sometimes several times per day.

Care continuity is also closely linked with the ERP systems. Our results suggest that if an ERP system did not consider teams when planning the schedules, the nurses experienced higher time pressure. In Finland, the use of ERP systems is intended to be a part of the management of older people care units, including home care [2]. If these systems or the work organization do not adequately prioritize or maintain team structures, including matching nurses with their own clients, then the nurses might have to care for a significant number of different clients. This can further lead to lower quality of care and higher time pressure, through ways discussed earlier. Previous study conducted in the Finnish home care services suggested that ERP systems might influence the job satisfaction and stress of the employees, as well as hinder the autonomy of work [4]. Considering the findings from previous studies and the present one, the exact role of ERP systems, how they can work together with self-organizing teamwork, and their relation to employee wellbeing should be further investigated.

Significant differences in time pressure were found between care teams with higher autonomy and teams with medium and lower autonomy, which indicates that nurses in teams with higher autonomy have lower time pressure. A similar finding was found in a study from Ruotsalainen and colleagues [23] among Finnish older people care nurses. These findings are important in the light of the previous studies considering self-organizing teams and employee wellbeing and satisfaction: If the nurses are not able to plan their day, or the client visits are planned for them without a possibility to influence the lists, this might lead to dissatisfaction. Nurses working in self-organizing teams have more autonomy and they are more satisfied with their work [26]. Being able to make independent decisions in teams has further made the work environment more attractive for nurses in self-organizing teams [44]. Therefore, our results suggest that team autonomy is an important factor when considering time pressure, and more generally wellbeing and job satisfaction of home care nurses. Due to the national guidance to use ERP systems in older people care, our findings can provide guidance on how to organize work on a team level, and they should there be taken into consideration when designing the ERP systems’ algorithms. In addition, despite the wide use of ERP systems, their influence on employee wellbeing remains largely unexplored in the context of Finnish home care.

The results from our study also indicated that the higher the proportion of breaks during the workday the lower the experienced time pressure. Previous studies suggest that breaktime is an essential factor in recovery from work [45] and in reducing stress [34], and high time pressure may indicate fewer or shorter breaks, which could eventually lead to poor recovery or stress. Additionally, there is evidence linking higher breaktime with lower turnover among nurses [34]. Our finding regarding lower breaktime and higher time pressure might suggest that there is flexibility missing from the home care nurses’ workday, which is important when considering the organization of work. The aim in the Finnish older people care policy has been to increase the direct care time up to 60% of the total workday [6]. However, if the work is not organized in a way that enables sufficient breaktime or the flexibility is low, the high intensity of the work may compromise nurses’ wellbeing. This can lead to further increases in time pressure, resulting in detrimental outcomes, such as lower care quality due to missed care [46] or higher turnover of personnel [47].

Additionally, in our study, if something disrupted the course of a workday, time pressure was perceived as higher among the respondents. As disruptions occur, nurses must prioritize which tasks they conduct during client visits, more so if the flexibility of work is low. This prioritization might lead to only performing ‘basic care’, while not being able to focus on the holistic care of a client [48]. Our findings indicated that the client’s care needs were not associated with the perceived time pressure. This outcome suggests that care needs are not necessarily a significant determinant of perceived time pressure, and instead, other factors, such as working conditions (e.g., frequent interruptions) or broader job demands, may play a more substantial role. This result may imply that clients with extensive care needs do not necessarily contribute to a heightened sense of time pressure, provided that sufficient time is allocated for the client visits and adequate support is available during the visit. This also implies that the care units have to some extent succeeded in taking care needs into account when the planning the length of care visits and the general division of work.

The results showed that higher age was independently associated with experiencing time pressure. This may be due to the physical nature of care work, where older staff members may require more time to perform tasks. Conversely, older employees may possess greater professional experience, enabling them to complete tasks more efficiently, which could, in turn, be associated with a reduced perception of time pressure. Although further studies are needed to explore this association. Furthermore, we found that if home care nurses experienced distress due to working alone (i.e., they experienced insufficient help and consultations possibilities, when needed), time pressure was perceived as higher. This might be simply related to a lack of staff, where there are no colleagues available when needed. Even though there is limited evidence regarding working alone and time pressure, previous studies have shown that working alone (i.e., without collegial support) has had negative effects on employee wellbeing in older people care [49]. Moreover, a study comparing the Nordic countries in terms of turnover intentions among care personnel in older people care services found that low support from a supervisor was associated with higher odds of turnover in all Nordic countries [50]. Supervisors in home care organizations should therefore make sure that they (or someone else) are available to provide consultation or support for employees. In addition, team meetings and other activities where difficult client cases, for example, can be discussed should be promoted actively in the work community. The results of this study thereby indicate that further attention should be paid to care continuity, team autonomy, workday flexibility, and use of ERP systems and how they fit into teamwork. Addressing these issues in home care management and work planning may both support work satisfaction and workforce retention as well as ultimately improve the quality of care for home care clients.

## Strengths and limitations

There were several limitations to this study. For instance, the study sample consisted of care units that participated on a voluntary basis; therefore, it is possible that participating units had better staffing levels compared to units that did not participate. However, the units were from different regions across Finland, and from both urban and rural areas. Still, generalization of the results to other countries or care settings should be done with caution. However, one of the strengths of this study was the high response rate, where despite the high effort related to filling up the time measurement forms, the nurses had also taken their time to complete the short wellbeing survey. In addition, we were able to assess the care workforce situation based only on managers’ self-assessed response, which may therefore be a very non-specific measure of the current situation, and somewhat subjective. Furthermore, the responses related to team autonomy and ERP systems were obtained from the managers’ survey, which provided information only from the organizational level, and therefore we only performed tests for comparing group means, where we were not able to adjusting for confounding variables. Using nurses’ views on autonomy as a dependent variable could have provided other, more subjective information related to team autonomy. The dataset used consisted of several sources of information, including subjective and indirectly measured job demands, in addition to RAI indicators. This can prevent common method variance, which may occur when using solely survey data [51]. To our knowledge, previous studies have not investigated the association between job demands (such as time pressure) and both subjective and objective variables using multiple sources of data. It is possible that other factors related to teamwork or leadership might have influenced the results of this study, however, these were not included in our survey, and thus could be important topics for future research. Lastly, this study was cross-sectional in nature and therefore it is not possible to determine any causality or directionality based on the findings.

## Conclusions

In order to decrease time pressure among home care nurses, factors related to job demands and organization of work need to be considered. Our results suggest that it is important to allow for flexibility in the nurses’ workdays and allocate enough time for breaks. In addition, Furthermore, if an ERP system is in use to schedule client visits, it should be designed to account for the team structure in order to secure care continuity, which is associated with better care quality. This approach may also help alleviate time pressure. Moreover, making changes to client lists should be possible if unexpected changes or disruptions occur. Due to the independent nature of home care work, sufficient collegial support is needed to cope with difficult situations. Time should be dedicated to team meetings, which can facilitate the self-organization of teams as well as opportunities to discuss complex client cases or challenging situations. Furthermore, home care teams should have more autonomy over their work, e.g., in planning of the client care or client visits, which might further lead to more satisfied staff and higher retention.

## Data Availability

The datasets generated and analyzed during this study are not publicly available but are available from the corresponding author on reasonable request.

## Abbreviations

CMI: Case Mix Index
ERP: Enterprise Resource Planning system
RAI: Resident Assessment Instrument
